# Infectious Disease: WNV Thrives in Financial Crisis

**Published:** 2009-01

**Authors:** Bob Weinhold

Criminal, civil, and journalistic investigators “follow the money” to identify the culprit of a crime. Public health detectives sometimes follow suit, since income is a well-known indicator of relative healthiness. In a new twist on this association, the worldwide financial crisis appears to be affecting human health in some settings, according to a team of researchers reporting in the November 2008 issue of *Emerging Infectious Diseases*. They studied home foreclosure and West Nile virus (WNV) incidence in the Bakersfield, California, area for 2006 and 2007 and found a parallel rise in both. Notices of delinquency rose 300%, soaring from 500 in the middle of 2006 to 1,500 in the middle of 2007. In the same period, documented human WNV cases rose 276%, peaking at 140.

The rise in WNV cases was a surprise, because the usual predictors—temperature, moisture, and infection rate and population of WNV hosts such as birds and vectors such as mosquitoes—initially pointed to a lower-risk period. The authors attribute the rise in WNV in part to a high number of abandoned homes with swimming pools, hot tubs, and ornamental ponds, many of which became breeding grounds for mosquitoes.

The correlation between abandoned water features and mosquito-borne diseases has been strongly suspected, but there had been little hard proof of the link. States with high foreclosure rates such as Arizona, California, and Florida are working to deal with problem pools. However, Rebecca Shultz, the Arthropod-borne Disease Surveillance Coordinator for the Florida Department of Health, points out that the California findings don’t currently apply to Florida because the latter state has had virtually no WNV for the past 3 years. One distinguishing factor may be that Florida’s surge in abandoned pools doesn’t add significantly to the total breeding area for mosquitoes, as the state is already naturally laced with water bodies, even during long-term drought.

Another factor may be a shift in California’s bird host makeup. William Reisen, the California study’s lead author and a research entomologist at the Center for Vectorborne Diseases at the University of California, Davis, says that persistent drought, WNV, and other diseases such as avian pox virus had contributed to the decimation of two important local WNV hosts, western scrub jays and house finches. But a third competent host, house sparrows, wasn’t hit as hard and rebounded strongly during 2007 with an abundance of young birds who are vulnerable to WNV.

As a followup to the study, Reisen’s team is working with the National Aeronautics and Space Administration, Google Earth, and four mosquito control districts representing diverse California settings to study abandoned pools and ponds, and to correlate their observed mosquito-breeding status with satellite images of the water bodies. Once this initial “ground-truthing” has been accomplished, satellite images themselves can provide a quick way to determine problem areas and circumvent typical barriers to effective surveillance such as climbing over fences around pools or relying on neighbors to report problems. If this works for California, Reisen anticipates the methodology could then be available for other states.

Although Florida isn’t currently in the same boat as California, Shultz says she is looking forward to having this new tool: “Any time you can speed up the timeline of getting data in, it’s very helpful.”

The interactions of natural and human forces in WNV and other emerging infectious diseases are complex, and much remains unknown. But Reisen says his team’s study, though limited in a number of ways, illuminates one small example of why such diseases are on the rise: the foreclosures, he says, are “just one aspect of anthropogenic factors altering the ecosystem.”

## Figures and Tables

**Figure:**
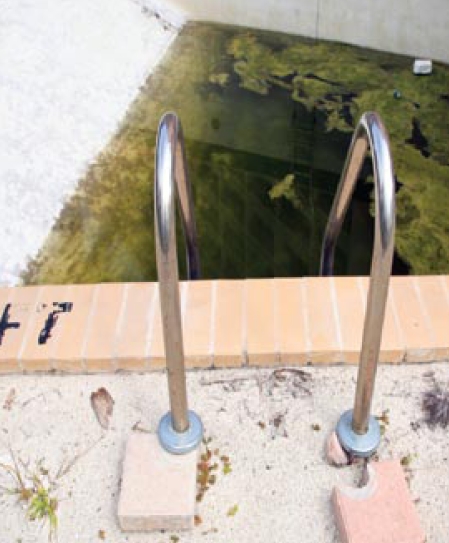
An abandoned pool is a move up for mosquitoes.

